# The performance of the K10, K6 and GHQ-12 to screen for present state DSM-IV disorders among disability claimants

**DOI:** 10.1186/1471-2458-13-128

**Published:** 2013-02-12

**Authors:** Bert LR Cornelius, Johan W Groothoff, Jac JL van der Klink, Sandra Brouwer

**Affiliations:** 1 Research Center for Insurance Medicine, Amsterdam, The Netherlands; 2 Department of Health Sciences, Community and Occupational Medicine, University Medical Center Groningen, University of Groningen, Groningen, The Netherlands; 3 Social Security Institute, Amsterdam, The Netherlands

**Keywords:** Disability, Mental disorder, Screening, CIDI, K10, K6, GHQ-12, Psychometric, Predictive value

## Abstract

**Background:**

Screening for mental disorders among disability claimants is important, since mental disorders seem to be seriously under-recognized in this population. However, performance of potentially suitable scales is unknown. We aimed to evaluate the psychometric properties of three scales, the 10- and 6-item *Kessler Psychological Distress Scale* (K10, K6) and the 12-item *General Health Questionnaire* (GHQ-12), to predict present state mental disorders, classified according to the *Diagnostic and Statistical Manual of Mental Disorders, 4*^*th*^*Edition* (DSM-IV) among disability claimants.

**Methods:**

All scales were completed by a representative sample of persons claiming disability benefit after two years sickness absence (n=293). All diagnoses, both somatic and mental, were included. The gold standard was the *Composite International Diagnostic Interview* (CIDI 3.0) to diagnose present state DSM-IV disorder. Cronbach’s α, sensitivity, specificity, positive (PPV) and negative predictive values (NPV), and the areas under the Receiver Operating Characteristic curve (AUC) were calculated.

**Results:**

Cronbach’s alpha’s were 0.919 (K10), 0.882 (K6) and 0.906 (GHQ-12). The optimal cut-off scores were 24 (K10), 14 ( K6) and 20 (GHQ-12). The PPV and the NPV for the optimal cut point of the K10 was 0.53 and 0.89, for the K6 0.51 and 0.87, and for the GHQ-12 0.50 and 0.82. The AUC’s for 30-day cases were 0.806 (K10; 95% CI 0.749-0.862), 0.796 (K6; 95% CI 0.737-0.854) and 0.695 (GHQ-12; 95% CI 0.626-0.765).

**Conclusions:**

The K10 and K6 are reliable and valid scales to screen for present state DSM-IV mental disorder. The optimal cut-off scores are 24 (K10) and 14 (K6). The GHQ-12 (optimal cut-off score: 20) is outperformed by the K10 and K6, which are to be preferred above the GHQ-12. The scores on separate items of the K10 and K6 can be used in disability assessment settings as an agenda for an in-depth follow-up clinical interview to ascertain the presence of present state mental disorder.

## Background

According to the Organization for Economic Co-operation and Development (OECD), poor mental health now accounts for one-third of all new disability benefit claims on average, rising to as high as 40-50% in some member states [1,2].

Despite their high prevalence, mental disorders often go unrecognized in health care settings [3-8], among workers [9-12] and among disability claimants [13]. A Dutch study in a cohort of persons with long term work disability due to mental health problems, mental disorders were found to be substantially under-diagnosed by social insurance physicians (IPs) assessing the disability benefit claim [13]. In a study (article submitted) of our own among disability claimants, we found very poor levels of agreement (kappa’s <0.260) between mental disorder certified by IPs and mental disorder classified according to the *Diagnostic and Statistical Manual of Mental Disorders, 4th Edition* (DSM-IV) [14], detected by the *Composite International Diagnostic Interview* (CIDI) [15] and, in a subgroup certified with a pure somatic disorder, the CIDI detected DSM-IV mood and anxiety disorder in 3.7% and 11.6% of cases, respectively. These findings are indications of serious under-recognition of mental disorder among disability claimants. In turn, the under-recognition of mental health problems in this group may lead to needs for treatment not being met, delayed return to work and unnecessary disability. Therefore, it is important that a reliable and valid screening instrument be made available for IPs for routine use in their assessment of disability benefit claims.

Most widely used short scales to screen for poor mental health are the *Kessler Psychological Distress Scale* with 10 (K10) or 6 items (K6) [16] and a short version of the *General Health Questionnaire* with 12 items (GHQ-12), adapted from Goldberg’s original 60-item GHQ [17]. These scales have been extensively used as screening tools in general population based studies, in primary care and in other samples of specific interest [18-24].

However, for several reasons, validity estimates for the K10, K6 and GHQ-12 observed in community samples, primary care and other populations may very well not be applicable in persons claiming disability after long-term sickness absence. In general the validity and optimal cut-off values of screening instruments in differentiating psychiatric cases from non-cases differ depending on the population in which the validity study is carried out, the golden standard that is used, the classification and the recall period of the disorders assessed and the method how to score screener responses. More specific, the prevalence of mental disorder in disability settings is much higher than in the general population and in primary care [25]. To add, studies have shown personal and environmental factors to interplay with mental health in sustaining long-term sickness absence and disability [26-28]. Therefore, in a population of disability claimants, validity of screening scales are likely to differ from those found in other populations. It is important to provide new information on the psychometric properties, including reliable cut-off values of the K10, K6 and GHQ-12 for use in this specific population. In the present study, we aim to determine the sensitivity, specificity and predictive power of these scales to detect any current DSM-IV psychiatric disorder in a population of disability claimants and to determine the optimal cutoff score of all scales.

## Methods

### Setting and procedures

In the Dutch social security system, one can apply for disability benefit after two years of continuous sick leave. Medical aspects of disability are then assessed by IPs employed by the Dutch Social Security Institute (SSI) in face-to-face interviews and examinations. For their assessment of diagnosis and treatment of the disorder(s) related to the disability claimed, IPs rely additionally in part on historic and actual medical data provided by occupational physicians who have assessed the sickness absence in the period preceding the disability claim. To classify diagnoses related to sickness absence and disability, IPs use a classification system derived from the ICD-10 and developed for use in occupational health and social security in the Netherlands [29]. The registry of the SSI allows one diagnosis code for any (somatic or mental) disorder as primary cause of disability, and two additional codes for any comorbid disorders as secondary or tertiary cause of disability.

For the present study, data were collected in the initial wave of a larger prospective cohort study with one year follow-up among disability claimants (PREDIS), conducted in the province of Groningen in the Netherlands. All persons claiming disability benefit at the SSI office in the city of Groningen in the period October 1st 2008 until January 1st 2010, were eligible to participate in the present study. As a result, all diagnoses were included, both mental and physical. The recruitment procedure was organised in two steps. As a first step, a SSI research assistant contacted eligible claimants by telephone asking permission to sent information about the study and a consent form. When permission was granted, name and address were given by the SSI assistant to the researcher, who then sent an information letter and a consent form as a second step. If eligible persons could not be contacted by telephone, the information letter and the consent form were sent by the SSI. Persons willing to participate returned signed consent forms to the researcher. The Medical Ethics committee of the University Medical Center Groningen (UMCG) approved recruitment, consent and field procedures.

Out of a total of 1544 eligible disability claimants, 375 persons participated in PREDIS after giving their informed consent prior to their inclusion in the study. The response rate is 24.3%. For the present study, we included 293 participants from whom we obtained complete data sets. Each participant was sent a questionnaire including the K10, with the K6 embedded, and GHQ-12. Subsequently, each respondent was face-to-face interviewed at home with the CIDI. Respondents returned completed questionnaires at the end of the interview.

To assess representativeness of the study sample for the target population, i.e. the national population of disability claimants in the Netherlands, we compared study data on prevalence of the most frequent ICD-10 defined mood, anxiety and stress-related disorders as primary cause of disability with a large national population (n=56.267) of all persons claiming disability benefit in the years 2006–2007 [2]. We found the study sample not to differ significantly from this national population, see Table 1.

**Table 1:** **Prevalence of ICD-10 defined mental disorders**^**a **^**in the study sample (n=293) and in the total population of disability claimants (n=56.267)**^**b**^

**ICD-10 category**	**Study sample n (%)**	**Population n (%)**	***p***^**c**^	**χ**^**2**^
Mood disorders	24 (8.2)	5.387 (10.2)	0.452	0.564
Anxiety disorders	15 (5.1)	2.668 (5.1)	0.730	0.119
Stress-related disorders	17 (5.8)	2.511 (4.8)	0.248	1.332
Total	56 (19.1)	10.566 (20.1)	0.491	6.423

^a ^Classified by IPs as primary cause for disability.

^b ^Disability benefit claimants in the Netherlands from Jan. 1^st^ 2006 to July 31^st ^2007 (source: SSI).

^c ^Proportions were tested with Chi-square goodness-of-fit test; P<0.05.

### Measures

#### K10 and K6

The 10-item Kessler Psychological Distress scale (K10) and its 6-item short-form the K6, measure non-specific psychological distress. Both scales have strong psychometric properties and are able to discriminate psychiatric cases from non-cases [8,19,21,23,30]. The K10 consists of 10 items with each five Likert-type response categories: ‘none of the time’ (1), ‘a little of the time’ (2), ‘some of the time’ (3), ‘most of the time’ (4) and ‘all of the time’ (5). Sum scores range from 10 to 50. The reference period of the K10 is 30 days. The K6 is a subset of the K10, using items 2, 4, 5, 8, 9 and 10 only, with sum scores ranging from 6 to 30. We used the official Dutch translation of the K10 [31].

#### GHQ-12

The 12-item General Health Questionnaire (GHQ-12) is a self-report instrument for the detection of mental disorders in the community and in primary care settings [24,32]. For the GHQ-12 we used the 0-1-2-3 scoring method with a four-point response scale: ‘not at all’ (for questions 1, 3, 4, 7, 8 and 12: ‘better than usual’) (0), ‘same as usual’ (1), ‘rather more than usual’ (2), ‘much more than usual’ (3) [24]. The reference period is the last few weeks. Sum scores range from 0 to 36. For the present study we used the Dutch version of the GHQ-12.

#### Gold standard: the Composite International Diagnostic Interview (CIDI)

As gold standard we used the Dutch translation of the CIDI, version 3.0 [15,33]. The CIDI is a comprehensive, fully-structured interview designed to be used by trained lay interviewers for the assessment of mental disorders according to the definitions and criteria of the DSM-IV. The validity of the CIDI 3.0 in assessing anxiety, mood and substance use disorders is generally good, as compared with clinical interviews [34]. Earlier CIDI versions also assess disorders with generally acceptable reliability and validity, with the exception of psychosis [35,36]. We included the sections Depression (D), Mania (M), Panic Disorder (PD), Specific Phobia (SP), Social Phobia (SO), Agoraphobia (AG), Generalized Anxiety Disorder (G), Suicidality (SD), Alcohol Use (AU), Illegal Substance Use (IU), Obsessive Compulsive Disorder (O), Psychosis Screen (PS), Post-Traumatic Stress Disorder (PT), Personality Disorders Screen (P), Attention Deficit Disorder (AD), Conduct Disorder (CD), Separation Anxiety Disorder (SA) and Interviewer’s Observation (IO). All respondents were face-to-face interviewed at their home. Interviewing was laptop computer-assisted. Mean interview time was 3 hours, but occasionally 5 to 6 hours, depending on the mental state of the respondent. For the present study, we used only DSM-IV Axis 1 disorders that occurred in the month preceding the interview (30-day diagnosis). This time frame corresponds with the recall period of the K10 and GHQ-12. Twelve CIDI interviewers (4 social insurance physicians, 2 medical students, 3 rehabilitation coaches, 3 insurance health secretaries) were trained by certified CIDI-trainers. Quality of interviewing techniques was evaluated bimonthly in group training sessions. Interviewers were blind to the classification of respondents to the K10 and GHQ-12.

#### Statistical analysis

We calculated the internal consistency (Cronbach’s alpha) of the K10, K6 and GHQ-12. An alpha coefficient of 0.70 or higher was considered to indicate good internal consistency. We analyzed the Receiver Operating Characteristic (ROC) [37] to calculate sensitivities, specificities, positive (PPV) and negative predictive values (NPV) for different cut-off values of all three scales in detecting any DSM-IV Axis I disorder that occurred in the last 30 days prior to the interview. Sensitivity is the probability that a person with the disorder is recognized by the test, while specificity is the probability that a person without the disorder is correctly recognized by the test. Positive predictive value (PPV) is the proportion of persons with true-positive test results. Negative predictive value (NPV) is the proportion of persons with true-negative test results.

We calculated the areas under the ROC curve (AUC) for all three scales with 95% confidence intervals. The ROC curve is a graphical plot of true positives (sensitivity) against the false positives (1-specificity) as the discrimination threshold (or cut-off point) is varied. The AUC equals the probability that a test will rank a randomly chosen respondent with a disorder higher than a randomly chosen respondent without a disorder. We defined as optimal cut-off score the value that gives the highest sum of the sensitivity and specificity, which is the point of the ROC-curve nearest to the upper left-hand corner of the graph. For the assessment of representativeness of the study sample for the target population, we used Chi-square goodness-of-fit test (P<0.05). For all statistical analyses we used SPSS version 16.0 for Windows.

## Results

### Sample characteristics

The study sample (n=293) comprised 154 female respondents (52.6%). The mean age was 50.0 (range 22–64). For further demographic characteristics as to educational level and urbanicity, see Table 2.

**Table 2:** Demographics and prevalence of present state DSM-IV disorders (n=293)

	**Total n(%)**
**Gender**	
Female	154 (52.6) ^a^
Male	139 (47.4)
**Age, mean (range)**	50.0 (22–64)
**Highest educational level**^**b**^	
Low	51 (17.6)
Intermediate	197 (67.9)
High	39 (13.4)
**Urbanicity**	
Rural (<10.000)	95 (32.4)
Midsize urban (10.000-100.000)	141 (48.1)
Urban (>100.000)	57 (19.5)
**Any (one or more) disorder**	76 (25.9)
**Any mood disorder**	30 (10.2)
Major depressive disorder	22 (7.5)
Minor depressive disorder	1 (0.3)
Dysthymia	15 (5.1)
Bipolar I/II disorder	8 (2.7)
(Hypo)mania	6 (2.1)
**Any anxiety disorder**	59 (20.1)
Panic attack	16 (5.5)
Panic disorder	5 (1.7)
Posttraumatic stress disorder	20 (6.8)
Social phobia	18 (6.1)
Agoraphobia	9 (3.0)
Specific phobia	21 (7.2)
Obsessive compulsive disorder	12 (4.1)
Generalized anxiety disorder	15 (5.1)
**Any substance use disorder**	8 (2.7)
Alcohol abuse	1 (0.3)
Alcohol dependence	2 (0.7)
Drug abuse	6 (2.0)
Drug dependence	3 (1.0)
**Other**	
Adult separation anxiety disorder^c^	4 (1.4)

^a ^Parenthetical numbers are percentages.

^b ^Low: elementary, preparatory middle-level; intermediate: middle-level applied; higher general continued; preparatory scientific; high: university applied sciences; research university; 6 cases are missing.

^c ^Adult Separation Anxiety Disorder is not listed in the DSM-IV.

In total, 76 participants (25.9%) met DSM-IV criteria for one or more 30-day mental disorder. Of this group, 49 participants (64.5%) had more than one mental disorder. The prevalence of any DSM-IV mood and any anxiety disorders was 10.2% and 20.1%, respectively, see Table 2. The 30-day prevalence of specific DSM-IV mental disorders in the study sample is also presented in Table 2. The median time between completing the K10, K6 and GHQ-12 and the CIDI was 4 weeks (SD: 5 weeks).

### Internal consistency

The internal consistency (Cronbach’s alpha) of all three scales used in the total sample (n=293) was good to excellent: 0.919 for the K10, 0.882 for the K6 and 0.906 for the GHQ-12.

### Sensitivity, specificity and predictive value

The AUC of the K10 for any 30-day DSM-IV disorder was 0.806 (CI 0.749-0.862), for the K6 0.796 (CI 0.737-0.854) and for the GHQ-12 0.695 (CI 0.626-0.765). Sensitivity, specificity, PPV and NPV for different cut-off scores of the K10, K6 and GHQ-12 for any 30-day DSM-IV disorder are presented in Table 3. The optimal cut-off score of the K10 was 24, of the K6 14 and of the GHQ-12 20 (see Table 3).

**Table 3:** **Sensitivity, specificity, positive predictive value (PPV) and negative predictive value (NPV) for different cut-off scores**^**a **^**of the K10, K6 and GHQ-12 for any present state DSM-IV disorder (n=293)**

**Score K10**	**Sensitivity**	**Specificity**	**PPV**	**NPV**
21	0.816	0.594	0.41	0.90
22	0.776	0.664	0.45	0.89
23	0.750	0.733	0.50	0.89
**24**	**0.724**	**0.779**	**0.53**	**0.89**
25	0.671	0.816	0.56	0.88
26	0.645	0.839	0.58	0.87
27	0.526	0.866	0.58	0.84
**Score K6**				
11	0.855	0.516	0.38	0.91
12	0.829	0.618	0.43	0.91
13	0.750	0.700	0.47	0.89
**14**	**0.684**	**0.770**	**0.51**	**0.87**
15	0.579	0.843	0.56	0.85
16	0.526	0.876	0.60	0.84
17	0.447	0.903	0.62	0.82
**Score GHQ-12**				
17	0.566	0.705	0.40	0.83
18	0.539	0.728	0.41	0.82
19	0.526	0.788	0.46	0.83
**20**	**0.487**	**0.829**	**0.50**	**0.82**
21	0.408	0.862	0.51	0.81
22	0.382	0.889	0.55	0.80
23	0.329	0.912	0.57	0.80

^a^ Optimal cut-off scores in bold letter type.

**Figure 1:**
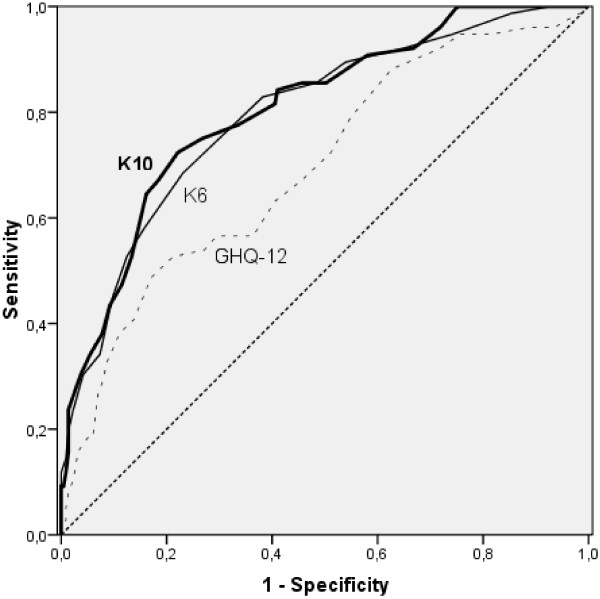
ROC curves for the K10, K6 and the GHQ-12 predicting any present state DSM-IV disorder.

Figure 1 shows the ROC-curves for all three scales predicting any 30-day DSM-IV disorder. In this graph, the dotted diagonal line represents the performance of a chance screener. All curves are located above this line of no information, indicating that all scales screen better than chance.

## Discussion

Our aim was to assess the sensitivity, specificity and predictive power of three short screening scales, the K10, its subset the K6 and the GHQ-12, to detect any present state DSM-IV mental disorder in a population of persons claiming disability benefit after two years of sickness absence. Our results show that all three scales have excellent Cronbach’s alpha’s. The K10 proved to be of good validity with an AUC of 0.806, while the AUC of the K6 is only marginally lower. In line with existing literature [20], both the K10 and the K6 seem to outperform the GHQ-12 as to validity. However, validity differences are statistically not significant, since confidence intervals overlap. The GHQ-12 may not be optimally suited for screening a population of long term disabled persons suffering from chronic mental health conditions. The GHQ-12 asks respondents to compare their present mental health, i.e. as experienced in the last few weeks, to their usual state and to indicate any changes. Therefore, persons with chronic poor mental health may respond that their present state is not different from their usual state. This may result in GHQ-12 scores that are too low.

We calculated an optimal cutoff score of 24 for the K10 (score range 10–50), 14 for K6 (score range 6–30) and 20 for the GHQ-12 (score range 0–40). These optimal scores are obtained by maximizing the sum of the sensitivities and the specificities of the three scales and represented by the points of the corresponding ROC-curves nearest to the upper left hand corner of the graph. However, in general, optimal cutoff values of a test are not determined by the outcome of simple statistics. They should be chosen after careful consideration, balancing costs and benefits that can be expected from correct and incorrect test outcomes [38]. However, in-depth analysis of expected costs and benefits of mental health screening is beyond the scope of this article. Instead, we show reliability data on the K10, K6 and GHQ-12 for different cutoff values. This allows physicians in insurance and occupational practice using these tests to choose the cut-off value that fits best their specific needs. For example, a practicing IP, using the K10 as mental health screener in an individual disability assessment and expecting unacceptable costs of a false-negative outcome for the claimant, may consider to choose a cut-off point lower than 24 we calculated as optimal cut-off score. If the claimant screens positive, the following clinical interview is likely to show without any further costs whether or not this positive screen result is false.

Since the psychometric properties of the GHQ-12 seem to be inferior to those of the K10 and the K6, we limit our discussion on how our validity findings compare to the literature to the K10 and the K6. We found the optimal cut-off score of the K10 to be 24 with sensitivity (SE):0.724 and specificity (SP): 0.779, and of the K6 to be 14 (SE: 0.684 and SP: 0.770). As we point out in the introductory section, it is difficult to compare the validity estimates we found for the K10 and K6 with those found in other studies, conducted in other populations, using other interviewing methods as golden standards, assessing different sets of DSM-IV classifications with different time-frames and using different scoring methods. The optimal cut-off value (24) we found for the K10 is higher than found by Donker et al. (2009) [8] in a Dutch primary care sample (optimal cut-off point 20; SE: 0.80; SP: 0.81) and by Fassaert et al. (2008) [23] in a general population sample of ethnic Dutch (optimal cut-off point 16.5; SE: 0.792; SP:0.768). It seems that in a population of disability claimants, the threshold for caseness is higher compared to the general population and primary care. This may primarily be based on population differences. First, it is well known that among long-term disabled persons psychosocial factors interplay with mental health related factors in sustaining long-term sickness absence and disability [26-28]. The importance of these psychosocial factors increase with the duration of sickness absence [26]. Therefore, distress found in the study sample may also be associated with psychosocial factors related to the sickness absence duration of two years, adding to the distress caused by the mental disorder itself. Second, the prevalence of mental disorder in our sample of disability claimants is much higher than found in other populations [39,40]. Although a higher prevalence does not systematically result in either higher or lower sensitivity and specificity, diagnostic test accuracy may vary with prevalence [41]. The study sample with a higher prevalence of mental disorder may include more severe disorders, resulting in higher cut-off scores for the K10. The optimal cut-off value (14) we found for the K6 almost equals the cut-off point found by Kessler et al. (2003) in a community sample, i.e. 13 (SE: 0.36 and SP: 0.96), while a higher cut-off point was to be expected. This may in our view primarily be explained by methodological differences: Kessler et al. used another structured psychiatric interview, assessing 12-month, not present state DSM-IV disorders and excluded substance-use disorders.

### Strengths and limitations

The strengths of this study are the use of the latest version of the CIDI, with almost complete covering of potential present state DSM-IV mental disorders, the employment of well trained interviewers, whose interviewing techniques were frequently evaluated and controlled, the use of three scales with proven reliability and validity in other research areas, and the representativeness as to mental health of the sample for the total population of disability claimants in the Netherlands.

The present study has some potential limitations. First, the response rate of 24.3% may have influenced the prevalence of mental disorders in the study sample by selection bias and, as a consequence, the external validity of the results. Predictive values of a test are strongly influenced by the prevalence of the condition under study. The low response rate in the present study may have resulted in selection bias in different ways. In general, persons suffering from mental illness might be less inclined to participate in surveys on mental health [33]. The low response may also be due to the stepped informed consent procedure, necessary to guarantee complete confidentiality and to prevent uninformed data flow between the researchers and the SSI. The same consent procedure was used in another Dutch study on mental health problems among long term work disabled persons [13]. The response rate in that study was comparably low: 25.8%. Finally, the low response rate in the present study may also be related to our measures, i.e. an extensive questionnaire and a lengthy psychiatric interview. The comprehensiveness of these measures may have kept eligible participants from giving consent. However, selection bias is less likely, since we found no significant difference as to the prevalence of most frequent mental disorders, i.e. mood, anxiety and stress-related disorders, diagnosed by the IPs in the study sample as compared to the national population of disability claimants. Second, the CIDI did not assess all possible DSM-IV diagnoses. Adjustment disorder, psychotic disorder, i.e. schizophrenia, and personality disorders cannot be diagnosed with the CIDI. Therefore, the use of the CIDI could have led to underestimation of prevalence of DSM-IV mental disorder in the study sample. Third, the median time interval between the questionnaire and the CIDI was 4 weeks, resulting in imperfect overlap of the recall periods of the scales and the time frame of the CIDI. Since mental health problems associated with long term disability are chronic conditions not likely to change in a short period of time, we believe that this imperfect overlap did not influence the validity of the scales in a significant way. To test this assumption, we compared the K10 and K6 sum scores with 12-month DSM-IV classifications present in the year preceding the interview. For both the K10 and the K6, we found validity estimates for 12-month classifications only to differ marginally from those for 30-day classifications, showing our assumption is likely to be right (K10: optimal cut-off point 23; SE: 0.649; SP: 0.842; AUC:0.798; K6: optimal cut-off point 13; SE: 0.746; SP: 0.771; AUC:0.787). Fourth, in theory it is possible that participants have overstated their mental complaints hoping to be considered for higher benefit. This may have resulted in a higher prevalence of mental disorders. However, in the information letter we sent to all eligible disability claimants, we stated explicitly that participation in the PREDIS cohort study would not influence the disability assessment by the SSI nor its outcome. Fifth, the questionnaire we administered to participants included the K10, with the K6 embedded. However, for analysis purposes the K10 and K6 were examined and reported on separately. It is possible that results could have been different had the K6 been administered as stand-alone. This means that any recommendation for use of the K6 as a stand-alone screening scale is cautionary.

## Conclusions

The K10 and K6 are reliable and valid instruments to screen for any present state DSM-IV disorder among disability claimants, with optimal cut-off scores of 24 for the K10 and 14 for the K6. The GHQ-12 has an optimal cut-off value of 20. The K10 and K6 are to be preferred above the GHQ-12. The K10 and the K6 are both very short scales and take only a few minutes to administer. While the validity of the K10 is slightly better than that of the K6, we advice to use the K10 instead of the K6 with cut-off values suitable for this particular population.

The scores on separate items of the K10 and the K6 can be used in disability assessments of long term sick listed workers as an agenda for an in-depth follow-up clinical interview to ascertain the presence of a present state mental disorder. By helping to identify concealed mental health problems and unmet needs for treatment in individual assessments, screening with the K10 or the K6 may be an important starting point of interventions to promote return to work and to prevent unnecessary long term disability, and may contribute to overall health improvement.

## Competing interests

The authors declare that they have no competing interests.

## Authors’ contributions

All authors participated in the design of the study and helped to draft successive concepts of the manuscript. BLRC drafted all concepts and the final manuscript, and performed the statistical analysis. All authors read and approved the final manuscript.

## Pre-publication history

The pre-publication history for this paper can be accessed here:

http://www.biomedcentral.com/1471-2458/13/128/prepub
